# Coordinated transcriptomic and metabolomic responses in rice reveal lignin-based physical barriers as key mechanisms of nonhost resistance to rust fungi

**DOI:** 10.1371/journal.pgen.1011679

**Published:** 2025-05-09

**Authors:** Ce Zhang, Liru Jian, Tao Guan, Yiping Wang, Huihui Pang, Yiqian Xu, Yaoyao Xing, Jiawen Wang, Zhensheng Kang, Jing Zhao

**Affiliations:** 1 College of Plant Protection, Northwest A&F University, Yangling, Shaanxi, People’s Republic of China; 2 State Key Laboratory for Crop Stress Resistance and High-Efficiency Production, Northwest A&F University, Yangling, Shaanxi, People’s Republic of China; 3 Key Laboratory of Plant Protection Resources and Pest Management, Ministry of Education, Yangling, Shaanxi, People’s Republic of China; Tsinghua University, CHINA

## Abstract

Nonhost resistance (NHR) serves as a fundamental defense response in plants against non-adapted pathogens, yet its underlying molecular mechanisms remain poorly understood. This study investigates the rice-*Pst* (*Puccinia striiformis* f. sp. *tritici*) interaction using integrated transcriptomic and metabolomic analyses to unravel the temporal dynamics of gene expression and metabolite changes associated with NHR. Our findings reveal a temporally coordinated activation of defense responses, with early induction of receptor-like kinases (RLKs) and hypersensitive response proteins, followed by later activation of jasmonic acid and systemic acquired resistance pathways, along with the accumulation of amino acids and other phenolic compounds. Notably, metabolic pathways related to cell wall reinforcement were significantly upregulated during *Pst* infection, highlighted by enhanced lignin biosynthesis (phenylpropanoid pathway), nucleotide sugar metabolism, and tryptophan pathways. Rice mutants deficient in genes involved in lignin biosynthesis (*OsPAL3*, *Os4CL3*, *Os4CL5*, and *OsCCoAOMT*) displayed reduced lignin deposition at infection sites and compromised resistance to *Pst*, underscoring a critical role of lignin-based physical barriers in NHR. This study provides novel insights into the molecular framework of rice NHR, emphasizing the pivotal role of structural defenses in plant immunity.

## Author summary

Our study reveals that rice nonhost resistance to *Pst* relies on coordinated transcriptional and metabolic responses, particularly highlighting lignin biosynthesis as a core defense mechanism. Early upregulation of RLKs and hypersensitive response proteins, followed by activation of systemic acquired resistance pathways, aligns with the accumulation of lignin and other compounds related to cell wall reinforcement, underscoring the role of structural barriers in nonhost immunity and providing new insights into the molecular framework of plant resistance to non-adapted pathogens.

## Introduction

Natural plants constantly face threats from various pathogens, including bacteria, fungi, virus, and nematodes. Despite this, plants in natural ecosystem generally remain healthy, with disease being the exception rather than the rule. This resilience is due to the specificity of most pathogens, which can only infect certain plant species (hosts) while being unable to infect others (nonhosts). This phenomenon, where all genotypes of a plant species resist to all genotypes of a potential pathogen species, is known as nonhost resistance (NHR) [1]. NHR is the most common defense mechanism protecting plants from most pathogens.

Nonhost responses to diverse non-adapted pathogens typically involves rapid immune responses, including reactive oxygen species (ROS) production, cell wall deposition, and localized cell death, resembling host resistance response. It is widely accepted that NHR is a result of multilayered defense, mediated by the interplay of pathogen-associated molecular patterns (PAMPs)-triggered immunity (PTI) and effector-triggered immunity (ETI) [2]. Vesicle trafficking mediated by SYP121 and EXO70B2 is crucial for forming defense structures such as papillae and encasements, which restrict filamentous pathogen invasion during nonhost interaction [3,4]. Additionally, recent studies showed that rice microRNAs enhance NHR against *Sclerotinia sclerotiorum* via cross-kingdom RNA interference [5]. Despite these insights, the molecular mechanisms underlying NHR remain largely unresolved.

The fungal pathogen *Puccinia striiformis* f. sp. *tritici* (*Pst*), the casual agent of wheat stripe rust, is among the most destructive wheat disease worldwide. As an obligate biotrophic pathogen, *Pst* has evolved a diverse arsenal of effector molecules that suppress host immunity by disrupting or interfering with critical components of the immune network [6–8]. Moreover, the strong selection pressure and frequent genetic variation in *Pst* have driven the emergence of new races capable of evading recognition by wheat resistance genes, ultimately causing recurrent epidemics. Over the past two decades, many stripe rust resistance genes have been rendered ineffective by these emerging *Pst* races. For instance, the resistance gene *Yr26*, widely deployed in Chinese wheat varieties, was overcome by the *Pst* race CYR34, which originated through genetic recombination [9]. Recently, new *Pst* isolates in China have also demonstrated virulence toward *Yr5* [10]. In this context, NHR represents a promising avenue for achieving durable and broad-spectrum resistance to wheat stripe rust.

Rice is the only gramineous crop that is recalcitrant to rust fungi [11]. Although *Pst* can germinate on rice leaf surface and attempt to penetrate leaf tissue through stomata under suitable conditions, most infection attempts are effectively halted at the infection sites, largely due to the production of H_2_O_2_ and callose deposition [12]. Studies have shown that traditional immune components, such as EDS1, RAR1, and CEBiP, do not contribute to rice’s NHR against *Pst* [13]. This suggests that rice’s NHR to *Pst* is likely governed by unknown mechanisms.

Lignin is a major aromatic polymer that constitutes the secondary cell wall in vascular plants [14]. It provides strength and imperviousness to cell walls, enabling long-distance water transport in vascular tissues [15]. Lignin is primarily composed of p-hydroxycinnamyl alcohols (monolignols), including p-coumaryl, coniferyl and sinapyl alcohols, which are oxidatively coupled to p-hydroxyphenyl (H), guaiacyl (G) and syringyl (S) lignin units, respectively. Monolignols are synthesized from phenylalanine via the phenylpropanoid pathway, involving numerous enzyme reactions, including phenylalanine ammonia-lyase (PAL), the first committed enzyme in phenylpropanoid pathway, and 4CL, which is responsible for the esterification of p-coumaroyl CoA to p-coumaric acid for lignin biosynthesis [16]. As a main component of the plant cell wall, lignin plays significant roles in plant growth and development by providing mechanical strength, rigidity, and hydrophobic properties. Additionally, lignin is critical in plant immune response to pests and pathogens. Pathogen-affected plants often show increased lignin accumulation in cell walls, forming a physical barrier against pathogen spread [17]. The expression of lignin biosynthesis genes is induced upon pathogen infection, leading to enhanced lignin accumulation at the infection sites [17–21]. Extensive studies suggest the defense function of lignin biosynthetic enzymes. Knockout or knockdown mutations in genes encoding monolignol biosynthetic enzymes result in immune deficiency. For example, silencing the genes encoding phenylalanine ammonia-lyase (TmPAL), caffeic acid O-methyltransferase (TmCAOMT), Caffeoyl-coenzyme A O-methyltransferase (TmCCoAMT), and cinnamyl alcohol dehydrogenase (TmCAD) in wheat (*Triticum monococcum*) increases susceptibility to *Blumeria graminis*, which causes powdery mildew [22]. However, it remains unclear whether lignin synthesis and deposition are involved in rice nonhost resistance against *Pst*.

In this study, we integrated comprehensive transcriptome and metabolome profiling analyses to identify genes and metabolites, regulatory networks, and pathways involved in rice nonhost response to *Pst* infection. The results demonstrated the involvement of the phenylpropanoid pathway (PPP) and tryptophan synthesis pathway in rice NHR to *Pst*, and highlighted the critical roles of lignin. Overall, this study provides a novel and multifaceted understanding of rice NHR to rust fungus.

## Results

### Transcriptomic reprogramming of rice during *Pst* infection

**Fig 1:**
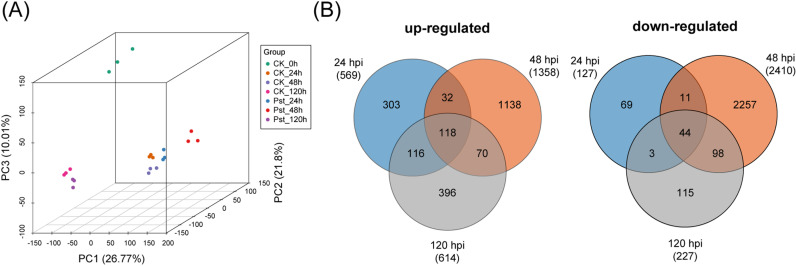
Transcriptomic analysis of rice genes regulated by *Puccinia striiformis* f. sp. *tritici* (*Pst*) infection. **(A)** Principal Component analysis (PCA) of the RNA-Seq samples from rice infected by *Pst*. **(B)** Venn diagram showing the distribution of upregulated and downregulated differentially-expressed genes (DEGs) across time points. Each RNA-Seq sample is represented as a dot in the PCA plot, visualized in three dimensions along the first three PCs. RPKM values from mock and *Pst*-inoculated samples were used for this analysis.

To investigate transcriptome alterations in rice response to *Pst* infection, leaf samples were collected from both infected and mock-inoculated plants at 0, 24, 48, and 120 hours post inoculation (hpi). RNA sequencing (RNA-Seq) was conducted, and the resulting reads were mapped to the Nipponbare reference genome (MSU v7). The number of reads obtained per sample ranged from 39.4 to 52.1 million, with 90.0%-97.1% mapping to the reference genome (S1 Table). Principal component analysis (PCA) was performed to assess variation across samples and evaluate the replicates based on gene expression profiles. The first two principal components (PCs) accounted for 48.6% of the total variation (Fig 1A). The three replicates of each sample clustered closely together, confirming dataset precision. Samples from the same time point clustered more closely than those from different time points, indicating a significant influence of inoculation conditions on gene expression patterns.

Differentially expressed genes (DEGs) between inoculated and mock samples were identified using thresholds of |log_2_(fold change)| ≥ 1 and FDR-adjusted *P*-value (padj) ≤ 0.05. At 24 hpi, 696 DEGs were identified (569 upregulated and 127 downregulated); at 48 hpi, 3,768 DEGs (1,358 upregulated and 2410 downregulated); and at 120 hpi, 841 DEGs (614 upregulated and 227 downregulated). Notably, only 32 genes were consistently upregulated, and 11 genes were downregulated across all time points (Fig 1B and S2 Table).

**Fig 2:**
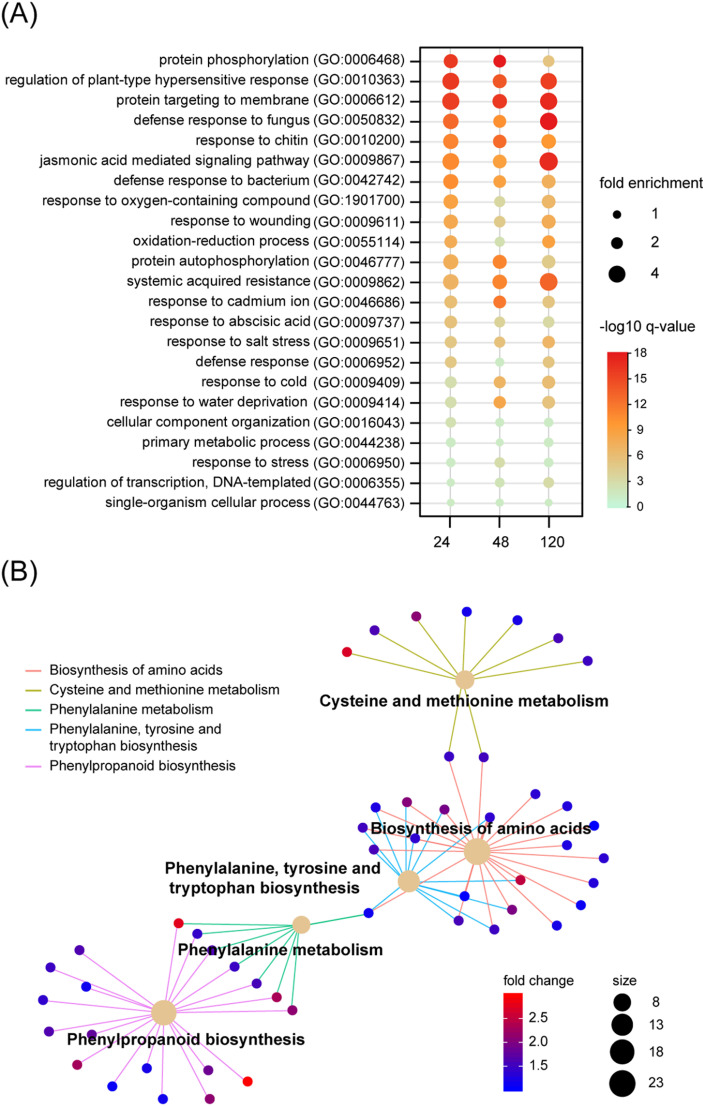
Analysis of metabolic pathway that differentially expressed genes (DEGs) involved. **(A)** GO enrichment of up-regulated DEGs in rice upon *Puccinia striiformis* f. sp. *tritici* infection among time points. Selected biological processes that are enriched among up-regulated DEGs from infected rice plants at 12, 24, and 120 hours post inoculation (hpi). The GO terms are listed along the y axis. The horizontal axis shows the time points. **(B)** Network plots the relationship of metabolic pathways enriched (FDR ≤ 0.05) by upregulated genes at 24 hpi. The colors indicate the comparison groups derived from RNA-Seq analysis.

To further elucidate the rice nonhost response to *Pst*, we conducted Gene Ontology (GO) enrichment and Kyoto Encyclopedia of Genes and Genomes (KEGG) pathway analyses. GO enrichment analysis of upregulated genes revealed distinct biological processes enriched at different time points. For example, “protein phosphorylation” was predominantly enriched at 24 and 48 hpi, while the “jasmonic acid mediated signaling pathway” showed significant enrichment at 120 hpi. Additional biological processes, including the “regulation of plant-type hypersensitive response”, “protein targeting to membrane”, “defense response to fungus”, “response to chitin”, “systemic acquired resistance”, were consistently enriched across all time points (Fig 2A). Notably, 130 predicted receptor like kinase (RLKs) were induced during rice-*Pst* interaction, including several important RLKs related to rice immunity, such as OsFLS2 (LOC_Os04g52780), OsBSR1 (LOC_Os09g36320), OsCRK6 (LOC_Os07g35690) [23], OsWAK91 (LOC_Os09g38850) [24], and SPL36 (LOC_Os12g08180) [25], as well as their partner such as CEBiP (LOC_Os03g04110) [26] (S2 Table). KEGG enrichment analysis identified significant upregulation at 24 and 120 hpi in pathways associated with phenylpropanoid biosynthesis, and phenylalanine, tyrosine, and tryptophan biosynthesis. At 48 hpi, upregulated genes were primarily enriched in pathways related to photosynthesis, starch and sucrose metabolism, and oxidative phosphorylation (Figs 2B and S1A–S1C and S3 Table).

MapMan analysis provide further insight into the metabolic pathways associated with DEGs at each time point during rice nonhost resistance to *Pst*. Pathways identified were consistent with those observe in the KEGG analysis. At 24 and 120 hpi, the most highly induced DEGs were related to secondary metabolism, including “Phenylpropanoids & Phenolics”, “Cell wall precursor synthesis”, and “Phe, Tyr, and Trp Synthesis”. At 48 hpi, upregulated genes were primarily associated with energy metabolism processes, such as “Light reactions” and “Starch metabolism”, whereas downregulated genes were linked to secondary metabolite processes, including “Terpenes”, “Flavonoids”, and “Phenylpropanoids & Phenolics” (S2A–S2C Fig).

### Metabolic changes of rice upon *Pst* Infection

To investigate metabolic pathways involved in the rice nonhost response to *Pst* infection, we conducted a wide-targeted metabolomics analysis. Metabolites from infected rice leaves at 48 hpi and 120 hpi, as well as from corresponding mock-inoculated leaves, were extracted and analyzed using HPLC-MS for both qualitative and quantitative assessments. Principal component analysis was performed to evaluate the reproducibility of measurement within biological replicates. The results demonstrated that biological replicates for each group consistently clustered together (S3 Fig), with over 75% of metabolites in all samples exhibiting a coefficient of variation (CV) value below 0.3 (S4 Fig) and a Pearson correlation coefficient (*r*) exceeding 0.8 (S5 Fig). These results validate the reliability and reproducibility of the metabolomic dataset.

**Fig 3:**
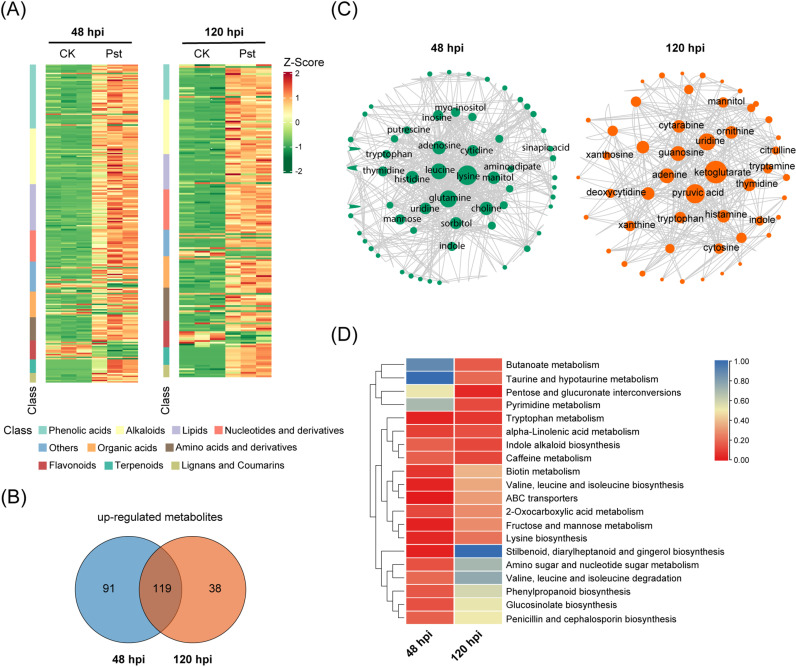
Metabolomic analysis of rice leaves infected by *Pst.* **(A)** Heatmap shows hierarchical clustering of class of differentially accumulated metabolites (DAMs) from metabolomics data of rice infected by *Puccinia striiformis* f. sp. *tritici* (*Pst*). Heatmap demonstrated z-scores obtained from enrichment analysis by using fold changes values and *P*-values from all the features detected from metabolomics. **(B)** Venn diagram indicating the distribution of up-regulated differentially-accumulated metabolites (DAMs) between 48 hpi and 120 hpi. **(C)** Metabolite–metabolite interaction networks highlighted the potential functional relationships among annotated DAMs at 48 hpi (green) and 120 hpi (orange). The chemical–chemical association for the metabolites network was extracted from STITCH. **(D)** Heatmap shows hierarchical clustering of the metabolic pathways from metabolomics data. Heatmap demonstrated z-scores obtained from enrichment analysis by using fold changes values and *P*-values from all the features detected from metabolomics. The colors indicated the significance as *P* values of enrichment for each metabolic pathway in rice.

A total of 1,133 metabolites were detected and significant differences were observed among groups (S4 Table). At 48 and 120 hpi, 210 and 157 metabolites were significantly increased, respectively, whereas only 24 and 21 metabolites showed significant decrease. Nearly all metabolite classes were induced upon *Pst* infection, except for flavonoids, where 11 and 8 metabolites were downregulated at 48 and 120 hpi (Figs 3A and S6), suggesting an overall strong induction of metabolites to combat fungi infection. Notably, 119 metabolites were consistently elevated at both 48 and 120 hpi, indicating a sustained metabolic response (Fig 3B).

To explore metabolite–metabolite interactions, we constructed a predictive network using STITCH 5 [27], highlighting potential functional relationships between detected metabolites and their associated pathways (Fig 3C). At 48 hpi, compounds related to amino acids metabolism, nucleotide metabolism, phospholipid metabolism, and lignin synthesis were influenced. By 120 hpi, nucleotide metabolism, amino acids, and energy metabolism were primarily impacted (Fig 3C). Differentially accumulated metabolites were largely enriched in pathways such as tryptophan metabolism, alpha-linolenic acid metabolism, indole alkaloid biosynthesis, and caffeine metabolism at both 48 and 120 hpi (Fig 3D and S5 Table). Specific enrichment was observed in ABC transporters pathways, stilbenoid, diarylheptanoid, and gingerol biosynthesis, as well as amino sugar and nucleotide sugar metabolism at 48 hpi (S7A Fig). In contrast, pathways like pentose and glucuronate interconversions, taurine and hypotaurine metabolism, and butanoate metabolism were exclusively enriched at 120 hpi (S7B Fig).

### Metabolic pathways related to cell wall reinforcement are strongly activated in response to *Pst* infection

We reasoned that the metabolic changes in *Pst*-infected rice were underpinned by alterations in gene expression. Therefore, we performed an integrated analysis of transcriptomic and metabolomics data. Finally, three pathways related to cell wall reinforcement were found to coincide substantially at both transcriptional and metabolic levels: the phenylpropanoids and phenolics pathway, the tryptophan and tryptamine synthesis pathway, and the nucleotide sugar metabolism pathway.

**Fig 4:**
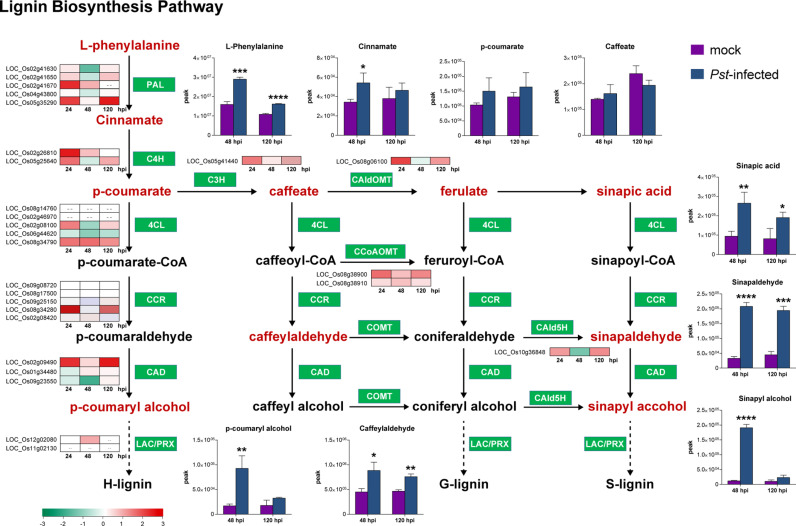
Integrative analysis of metabolomics and transcriptomics revealing activation of lignin biosynthesis pathway in rice infected by *Pst.* A schematic of the lignin synthesis pathway showing the relative levels of specific metabolites in rice leaves infected with *Puccinia striiformis* f. sp. *tritici* (*Pst*) (blur bar) or mock-inoculated leaves (purple bar) at 48 and 120 hours post inoculation (hpi), and the relative transcription levels of selected genes at 24, 48, and 120 hpi. Metabolite peak intensities (n = 3, average ± SD) are depicted on the primary y axis. Asterisks indicate statistically significant differences compared to the mock controls using two-sided Student’s *t*-test. (*P < 0.05, **P < 0.01). The heatmap represents changes in transcript level of genes as log_2_(fold change) in comparison with the mock controls. Enzymes catalyze the biochemical reaction are indicated in white text in green box. PAL, phenylalanine ammonia lyase; C4H, cinnamate 4-hydroxylase; 4CL, 4-coumarate-CoA ligase; CCR, cinnamoyl-CoA reductase; CAD, cinnamyl alcohol dehydrogenase; LAC, laccase; C3H, p-coumaroyl ester 3-hydroxylase; CAIdOMT, 5-hydroxyconiferaldehyde O-methyltransferase; CCoAOMT, caffeoyl-CoA O-methyltransferase; CAId5H, coniferaldehyde 5-hydroxylase.

The phenylpropanoid biosynthesis pathway (ko00940) was notably activated, with significant induction of key enzymes and metabolites, particularly in the lignin biosynthesis branch. Genes encoding critical enzymes in this pathway, including phenylalanine ammonia lyase (PAL), cinnamate 4-hydroxylase (C4H), p-coumaroyl ester 3-hydroxylase (C3H), 4-coumarate CoA ligase (4CL), cinnamoyl-CoA reductase (CCR) and cinnamyl alcohol dehydrogenase (CAD), as well as caffeoyl-CoA O-methyltransferase (CCoAOMT), coniferaldehyde 5-hydroxylase (CAld5H) and 5-hydroxyconiferaldehyde O-methyltransferase (CAldOMT), were all upregulated to varying degrees (Figs 4 and S8). Correspondingly, intermediates in the phenylpropanoid biosynthesis pathway showed significant increase upon *Pst* infection, with higher induction at 48 hpi compared to 120 hpi. Among the 145 predicted peroxidases in rice [28], which are responsible for the final step of lignin synthesis from monolignol, 12, 4, and 10 were induced at 24, 48, and 120 hpi respectively (S6 Table). However, the transcriptional levels of most genes encoding laccase [29], another enzyme involved in the final step of lignin synthesis, remained unchanged across all three time points (S7 Table). Interestingly, genes involved in the flavonoid synthesis, another branch of the phenylpropanoids pathway, such as chalcone synthase (CHS), chalcone isomerase (CHI), flavone synthase II (FNSII), flavonoid 3’-hydroxylase (F3’H), flavonoid O-methyltransferase (FOMT) and chrysoeriol 5’-hydroxylase (C5’H), showed no significant transcriptional changes, with resultant fewer flavonoids exhibited elevated levels compared to controls.

**Fig 5:**
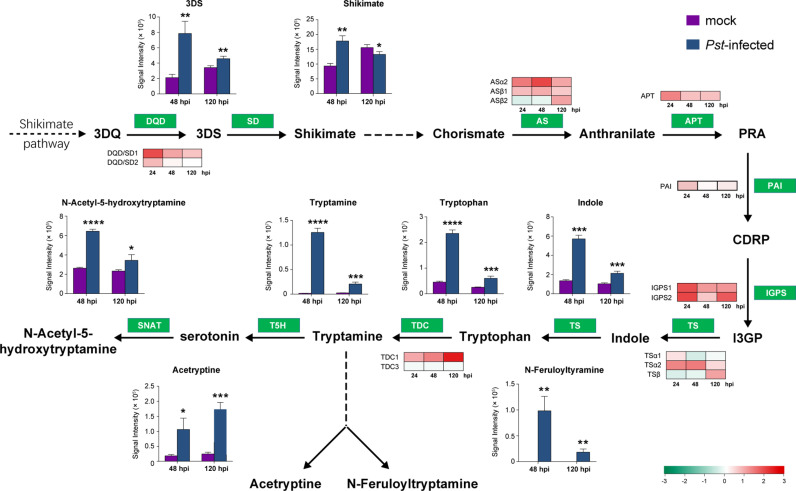
Integrative analysis of metabolomics and transcriptomics revealing activation of tryptophan and derivatives biosynthesis pathway in rice infected by *Pst.* A schematic of the lignin synthesis pathway showing the relative levels of specific metabolites in rice leaves infected with *Puccinia striiformis* f. sp. *tritici* (*Pst*) (blur bar) or mock-inoculated leaves (purple bar) at 48 and 120 hours post inoculation (hpi), and the relative transcription levels of selected genes at 24, 48, and 120 hpi. Metabolite peak intensities (n = 3, average ± SD) are depicted on the primary y axis. Asterisks indicate statistically significant differences compared to the mock controls using two-sided Student’s *t*-test. (*P < 0.05, **P < 0.01). The heatmap represents changes in transcript level of genes as log_2_(fold changes) in comparison with the mock controls. Enzymes catalyze the biochemical reaction are indicated in white text in green box. 3DQ, 3-dehydroquinate; 3DS, 3-Dehydroshikimate; PRA, N-(5-Phospho-beta-D-ribosyl)-anthranilate; CDRP, 1-(2-Carboxyhpenylamino)-1’-deoxy-D-ribulose 5-phosphate; I3GP, indole-3-glycerol phosphate; DQD, 3-dehydroquinate dehydratase; SD, shikimate dehydrogenase; AS, anthranilate synthase; APT, anthranilate phosphoribosyltransferase; PAI, PR-anthranilate isomerase; IGPS, indole-3-glycerol phosphate synthase; TS, tryptophan synthase; TDC, tryptophan decarboxylase; T5H, tryptamine 5’-hydroxylase; SNAT, serotonin N-acetyltransferase.

Tryptophan is not only an essential amino acid but also serve as an intermediate in the biosynthesis of indole alkaloid, such as tryptamine and serotonin, which play vital roles in plant defense. The levels of 3-dehydroshikimate (3DS) and shikimate, the first two intermediates of tryptophan pathway synthesized from 3-dehydroquinate (3DQ) by DQD, were significantly increased at 48 hpi. Consistently, DQD1 transcript levels, responsible for converting 3DQ to shikimate, were upregulated in inoculated samples from 24 hpi and remained relative high at 48 hpi and 120 hpi. The DQD2 transcript was also induced at 24 hpi but showed minimal change at later time points compared to controls. Additionally, transcripts for nearly all enzymes involved in the tryptophan synthesis pathway were induced during the nonhost interaction (Fig 5). These enzymes include anthranilate synthases (AS), which convert I3GP to tryptophan. As the results, tryptophan and indole levels increased after *Pst* infection, particularly at 48 hpi. Furthermore, the transcript level of TDC1, one of the two enzymes responsible for the conversion of tryptophan into tryptamine, was also drastically induced upon the *Pst* infection, accompanied by a significant elevation in tryptamine level. Thus, both enzymes and intermediates in the tryptophan synthesis pathway were strongly induced, promoting the production of tryptophan derivatives such as indole alkaloids. Tryptophan biosynthesis originates from the shikimate pathway in common with the biosynthesis of phenylalanine—the primary precursor of lignin biosynthesis. Thus, activation of the shikimate pathway enhances the synthesis of both tryptophan and lignin.

**Fig 6:**
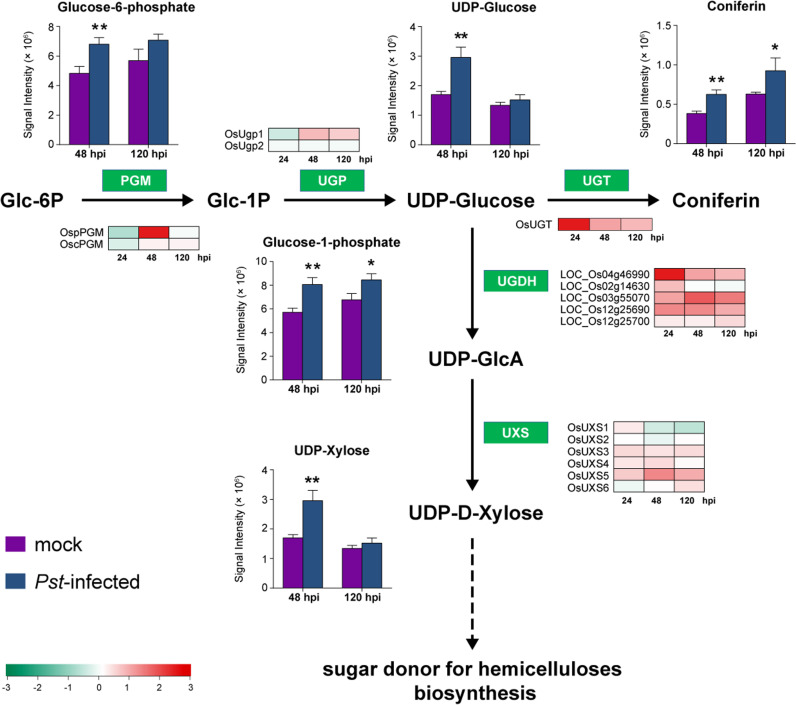
Transcript and metabolite changes in the nucleotide sugar pathway in rice infected by *Pst.* A schematic of the nucleotide sugar biosynthesis pathway showing the relative levels of specific metabolites in rice leaves infected with *Puccinia striiformis* f. sp. *tritici* (*Pst*) (blur bar) or mock-inoculated leaves (purple bar) at 48 and 120 hours post inoculation (hpi), and the relative transcription levels of selected genes at 24, 48, and 120 hpi. Metabolite peak intensities (n = 3, average ± SD) are depicted on the primary y axis. Asterisks indicate statistically significant differences compared to the mock controls using two-sided Student’s *t*-test. (*P < 0.05, **P < 0.01). The heatmap represents changes in transcript level of genes encoding enzymes involved in nucleotide sugar metabolism as log_2_(fold change) in comparison with the controls. Enzymes catalyze the biochemical reaction are indicated in white text in green box. Glc-6P, Glucose 6-phosphate; Glc-1P, Glucose-1-phosphate; UDP-GlcA, UDP-glucuronic acid; PGM, phosphoglucomutase; UGP, UDP-glucose pyrophosphorylases; UGT, UDP-glucosyl transferase; UGDH, UDP-glucose-6-dehydrogenase; UXS, UDP-glucuronic acid decarboxylase.

The nucleotide sugar biosynthesis pathway was significantly regulated during the rice nonhost defense response to *Pst* infection (Fig 6). The level of glucose-6-phosphate, the starting molecule in the nucleotide sugar biosynthesis pathway, increased at 48 hpi, followed by an increase in glucose-1-phosphate at both 48 hpi and 120 hpi. As expected, the level of UDP-glucose, a key intermediate in the nucleotide sugar pathway, was significantly high at 48 hpi compared to the mock control. As a result, the final product, UDP-D-Xylose—a sugar donor for hemicelluloses biosynthesis, was elevated, was along with Coniferin, a lignin precursor converted by glucose-6-phosphat [30,31]. Consistent with the accumulation of these compounds, transcripts for genes involved in nucleotide sugar biosynthesis, including *PGM*, *UGT*, *UGDH*, and *UXS* were upregulated upon *Pst* infection (Fig 6). Therefore, activation of the nucleotide sugar biosynthesis pathway not only enhances cell wall polysaccharide production but also promotes lignin biosynthesis. These findings suggest that cell wall components, such as lignin, may play critical role in rice nonhost resistance to *Pst*.

### Mutation of genes involved in the lignin synthesis pathway significantly attenuates rice nonhost resistance to *Pst*

To validate the role of lignin in rice nonhost resistance to *Pst*, we assessed rice mutants with disrupted lignin synthesis genes for their resistance response to *Pst* infection. Four genes, *OsPAL3* (*LOC_Os02g41670*), *Os4CL3* (*LOC_Os02g08100*), *Os4CL5* (*LOC_Os08g34790*), and *OsCCoAOMT* (*LOC_Os08g38900*), which are crucial for lignin synthesis and showed upregulation upon *Pst* infection were selected. *OsPAL3* encodes phenylalanine ammonia-lyase, the first enzyme in the phenylpropanoid pathway, while *Os4CL3* and *Os4CL5* encodes the 4-coumarate-CoA ligase, an enzyme essential for converting p-coumarate, caffeate, and ferulate into their corresponding CoA esters in lignin biosynthesis. Among the five 4CL isoforms characterized in rice [32], *Os4CL3* was up-regulated at 24 hpi and *Os4CL5* exhibited consistent induction during *Pst* infection. CCoAOMTs catalyze the conversion of caffeoyl-CoA into into feruloyl-CoA.

**Fig 7:**
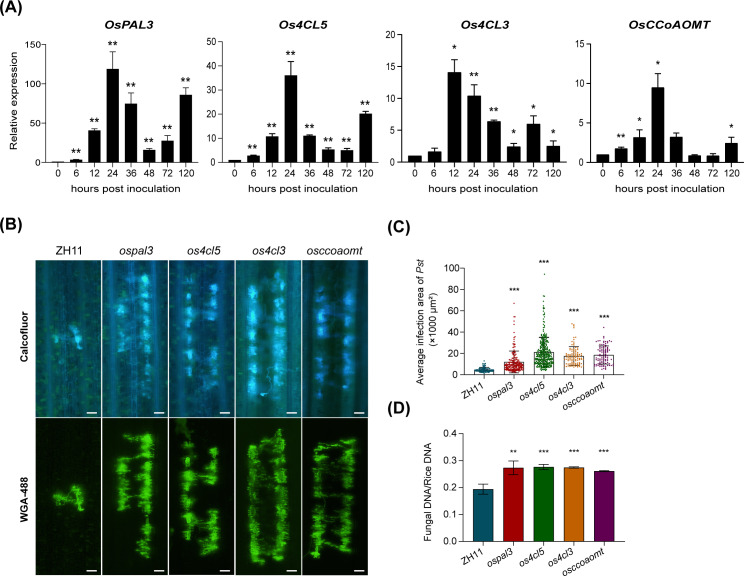
Mutation of genes involved in the lignin synthesis pathway significantly attenuates rice nonhost resistance to *Pst.* **(A)** The relative transcription pattern of *OsPAL3*, *Os4CL5*, *Os4CL3*, and *OsCCoAOMT* in rice infected by *Puccinia striiformis* f. sp. *tritici* (*Pst*). The relative expression levels were determined using the comparative threshold method (2^-ΔΔCT^) and normalized to *OsActin1*. Values represent means from three biological replicates, with error bars indicating the standard deviation (SD). Asterisks indicate statistically significant differences compared to samples at 0 hours post inoculation (hpi) with *Pst* using two-sided Student’s *t*-test (*P < 0.05, **P < 0.01). **(B)** Histological observations of *Pst* development in rice leaves from wild type and mutant plants at 14 dpi. Fungal structures were visualized using Calcofluor and WGA-488 staining respectively, and observed with a fluorescence microscope. Scale bars: 50 μm. **(C)** Infection site areas at 14 dpi (in units of µm^2^). Data are shown as means ± SD. Differences were assessed using a two-sided Student’s *t*-tes*t* (compared to the ZH11, ***P < 0.001). **(D)** Biomass ratio (*Pst*/rice) of total DNA extracted from wild-type (ZH11) and mutant rice leaves at 14 dpi. The ratio of total fungal DNA to total rice DNA was assessed using the *Pst* gene *PstEF1* and the rice gene *OsActin1* (*LOC_Os03g50885*). Data are shown as means ± SD from three biological replicates. Differences were assessed using a two-sided Student’s *t*-test (compared *t*o the ZH11, **P < 0.01 and ***P < 0.001).

The induction of four lignin biosynthesis related genes during rice-*Pst* interaction was validated by qRT-PCR. The transcriptional levels of all these genes were induced and peaked at 24 hpi or 12 hpi (Fig 7A). The corresponding mutants for these genes were identified from a rice CRISPR-Cas9 mutant database (S9 Fig) [33]. The resistance of these mutants was evaluated by inoculating homozygous seedlings with *Pst*. Compared to wild-type rice (ZH11), the mutants exhibited larger *Pst* colonies in leaf tissue at 14 dpi (Fig 7B and 7C and S8 Table). Additionally, a higher ratio of *Pst* biomass to rice in the mutants confirmed increased fungal growth, indicating a reduction in nonhost resistance in these mutants (Fig 7D and S9 Table).

**Fig 8:**
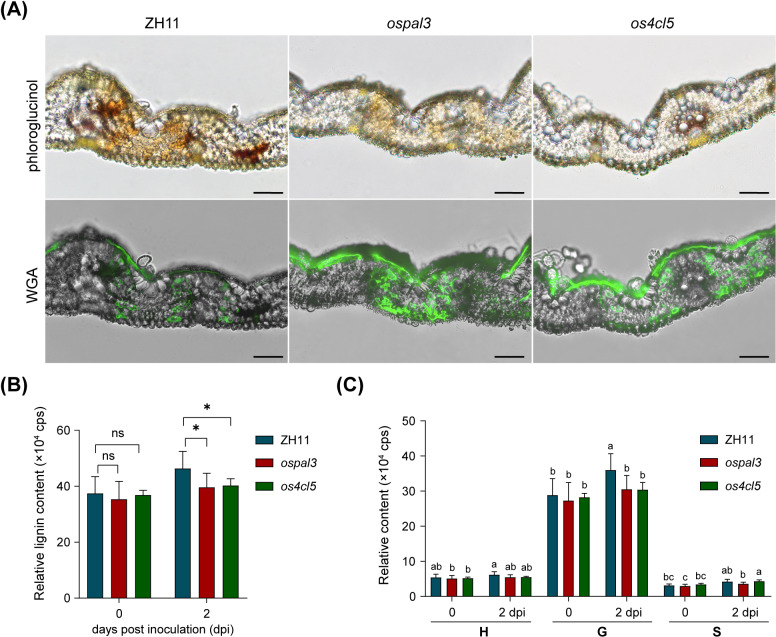
Reduced lignin accumulation at *Pst* infection sites in rice mutants for *OsPAL3* and *Os4CL5.* **(A)** Histochemical analysis of leaf sections from *ospal3*, *os4cl5* mutants, and wild-type (ZH11) plants at 14 days post inoculation (dpi) with *Puccinia striiformis* f. sp. *tritici* (*Pst*). The reddish-brown intensity of phloroglucinol-HCl staining indicates lignin content, and the green fluorescence from wheat germ agglutinin (WGA) staining highlights *Pst* infection sites. Scale bars: 50 μm. **(B)** Quantification of lignin contents in leaves from rice mutants *ospal3* and *os4cl5* before or after inoculated with *Pst*. Data are shown as means ± SD (n = 5; 5–10 leaves each). Statistically significant differences are indicated with asterisks (Student’s t-test, *P < 0.05). **(C)** Relative contents of lignin monomers. Different letters indicate significant differences (Tukey’s HSD test; P < 0.05).

To examine whether the reduced resistance of *ospal3* and *os4cl5* mutants was due to the impaired lignin synthesis, lignin accumulation in infected rice leaves was visualized using phloroglucinol staining (Fig 8A). In wild type rice, distinct reddish-brown staining appeared near infection sites (indicated by green fluorescence of fungi marked with WGA488), suggesting that *Pst* infection triggers lignin production in rice cells. In contrast, only faint lignin staining was observed near infection sites on mutant leaves. Quantification of lignin contents further confirmed defective lignin biosynthesis in the mutants (Fig 8B). Additionally, analysis of lignin monomer composition revealed that the reduction in lignin was primarily resulted from decreased levels of coniferyl alcohol (monolignol G) (Fig 8C and S10 Table). These findings demonstrate that mutations in *OsPAL3* and *Os4CL5* compromise lignin production during *Pst* infection, reducing nonhost resistance in rice and underscoring lignin’s crucial role in rice defense against stripe rust fungus.

## Discussion

NHR is known to be a common defense response of the plant immune system [1]. Previous studies have characterized rice NHR against *Pst* from genetic and histological perspectives [12,34]. However, the mechanisms of nonhost resistance in regulating gene expression and the associated changes in metabolites remain poorly understood. In this study, we systematically investigated the changes in gene expression and metabolite profiles through an integrated of metabolomic and transcriptomic datasets, to elucidate rice nonhost resistance triggered by *Pst* infection.

We have clearly shown that the rice-*Pst* interaction induces temporally coordinated patterns of gene expression. This analysis presents a detailed landscape of convergence and divergence across different time points in rice transcriptomic responses to rust fungus infection. We observed a significant enrichment of genes related to the regulation of hypersensitive response and protein targeting to membrane across all three time points. Notably, proteins involved in early defense signaling pathways, such as receptor-like kinases (associated with protein phosphorylation), were specifically enriched at 24 and 48 hpi. Conversely, genes involved in the jasmonic acid (JA) signaling pathway and systemic acquired resistance (SAR) showed a more substantial enrichment at 120 hpi compared to 24 or 48 hpi.

A substantial number of receptor like kinase (RLKs) were upregulated during rice-*Pst* interaction. RLKs plays a critical role in signal perception, acting as key upstream components in plant defense signaling pathways [35]. While RLKs are primarily responsible for recognizing pathogen-associated molecular patterns and initiating immune signaling, their transcription is also modulated by positive feedback mechanisms [36]. For instance, transcriptional induction of *FLS2* by flg22 is diminished in ethylene-insensitive mutants, indicating a positive feedback regulation of *FLS2* transcripts through flg22-induced ethylene production [37]. Moreover, the extensive upregulation of several functionally identified RLKs implies that *Pst* infection activates multiple disease resistance pathways in rice. This likely contributes to the robustness and durability of rice’s nonhost resistance.

Our joint analysis of RNA-seq and metabolomics provided clear evidence of coordinated changes in rice metabolic pathways, particularly those involved in the biosynthesis of aromatic amino acids. In addition to being incorporated into proteins, amino acids are precursors for a diverse range of specialized metabolites, such as signaling molecules, phytoalexin, and the cell-wall component. The critical role of amino acid metabolism in plant immunity has gained attention in recent years [38]. In particular, amino acids like glutamine, lysine and alanine were accumulated in rice leaves treated with chitin and flg22 [39]. Additionally, metabolomic analyses of plants infected by various pathogens have reported amino acid accumulation [40–42]. Nine amino acids accumulated in response to *Pst* infection. Notably, our findings highlight the pivotal role of tryptophan (Trp) in rice NHR. Trp serves as a precursor for key indolic defense compound, including camalexin, indolic glucosinolates, and indole-3-acetic acid [43,44]. Trp-derived secondary metabolites have been extensively linked to NHR in Arabidopsis, particularly against *Plectosphaerella cucumerina* and *Phytophthora brassicae* [45,46].

Beyond its role in chemical defense, Trp-derived metabolites contribute to cell wall fortification. Serotonin, a Trp derivative, is integral to rice’s physical defense against *Bipolaris. oryzae* through its role in forming structural barriers [47,48]. Upon *Pst* infection, several Trp-derived metabolites, including tryptamine, N-Acetyl-5-hydroxytryptamine, acetryptine, and N-feruloyltryptamine, accumulated significantly, while the levels of serotonin remained unchanged. This suggest promptly serotonin oxidization by pathogen-induced ROS or its conversion to melatonin. Supporting this, we observed a marked increase in N-acetyserotonin (N-Acetyl-5-hydroxytryptamine), a precursor of melatonin. Recent studies indicate that melatonin confers cotton resistance against *Verticillium dahlia* by modulating lignin and gossypol synthesis genes expression [49,50]. Thus, activation of the tryptophan biosynthesis pathway likely also contribute to the physical defense to *Pst* infection.

The phenylpropanoid pathway serves as a starting point for the biosynthesis of numerous essential secondary metabolites in plants, including flavonoids (such as anthocyanin, proanthocyanidin, and flavonols), coumarins, and lignins [51]. Extensive studies have demonstrated the crucial roles these compounds play in plant defense [52]. Our analysis shows the enrichment of DEGs related to phenylpropanoid metabolism, along with the substantial accumulation of phenolic acids, lignans, and coumarins in rice-*Pst* interaction, highlights the importance of the phenylpropanoid pathway. Notably, while the lignin pathway—a branch of the phenylpropanoid pathway—was activated during *Pst* infection, the flavonoid biosynthesis branch was downregulated, as indicated by the decreased expression of *CHS* (*LOC_Os11g32650*) and *CHI* (*LOC_Os03g60509*), two key flavonoid biosynthetic genes, and reduced abundance of most detected flavonoids.

In contrast, the activation of the lignin biosynthesis pathway during *Pst* infection suggests a key defensive role for lignin. Lignin is a complex polymer that forms a physical barrier against pathogen invasion due to its resistance to microbial degradation [53]. Consistent with the accumulation of monolignin, histological staining confirmed increased lignin deposition around the *Pst* infection sites. Moreover, rice mutants for *OsPAL3* and *Os4CL5* exhibited reduced lignin deposition at infection sites and decreased resistance to *Pst*, further supporting a model in which nonhost resistance to *Pst* relies primarily on physical barriers. Our previous studies also found an absence of host cell death during the initial stages of rice-*Pst* interaction [34], suggesting that rice nonhost resistance is largely composed of pre-penetration resistance. In fact, lignin production as a fundamental defense mechanism for cell wall reinforcement has been noted in diverse host-pathogen interactions, including wheat and *Pst* interaction [54]. Temporal silencing *TaPAL* in wheat compromises resistance to an avirulent *Pst* race, highlighting the role of lignin biosynthesis in wheat defense against *Pst* [55].

In addition to lignin abundance, lignin composition may contribute to the differential output of defense response between rice and wheat [56,57]. Metabolomic results indicate a distinctive activation of sinapyl alcohol (S-lignin) and p-coumaryl alcohol (H-lignin) biosynthesis, compared to conifer alcohol (G-lignin). However, rice mutants in *OsPAL3* and *Os4CL5* compromised in NHR demonstrated lower levels of G-lignin in infection sites. Thus, variations in lignin composition may result from differences in genes involved in the lignin biosynthesis pathway and their expression patterns.

## Materials and methods

### Plant materials and *Pst* inoculation

Rice seedlings of japonica cultivar Nipponbare were grown under a 16/8 hours photoperiod cycle, at day/night temperatures of 26/22°C and a relative humidity of 60% in growth chambers. Three-week-old plants at the three-leaf stage were inoculated using *Pst* isolate Fxc-7 and placed in 12°C with 100% humidity for 36 hr. For inoculation of rice, 10 μl fresh *Pst* urediniospores suspensions (50 mg urediniospores ml^-1^ in 3M Novec 7500 Engineered Fluid) were applied with a pipette onto the adaxial surface of the second leaf from 3-week-old rice seedlings. Rice leaves inoculated or treated by Novec 7500 (as mock control) were sampled at for RNA and metabolites extraction. For RNA extraction, leaves from 5 individual plants inoculated or mock-inoculated at 0, 24, 48 and 120 hpi were collected and pooled for each condition. For metabolites, leaves from 30 individual plants inoculated or mock-inoculated at 0, 48 and 120 hpi were collected and pooled for each condition.

The rice CRISPR-Cas9 mutants for lignin biosynthesis related genes were obtained from the RGKO library [33].

### RNA sequencing and transcriptome data analysis

Leaf samples were frozen in liquid nitrogen and ground to a fine powder using a mortar and pestle. Total RNA was extracted using TRIzol (Invitrogen) according to the manufacturer’s instructions. A total amount of 1 μg RNA per sample was used as input material for the RNA sample preparations. Sequencing libraries were constructed using NEBNext Ultra RNA Library Prep Kit for Illumina (NEB, USA) following manufacturer’s recommendations. Deep sequencing was performed using an Illumina NovaSeq 6000 for 150 paired-end sequencing. By removing reads containing adapter, reads containing ploy-N and low quality reads from the raw data, the clean sequence reads were obtained and mapped to the Nipponbare cDNA reference database from Rice Genome Annotation Project v7.0 (RGAP; http://rice.uga.edu/) using the HISAT2 [58]. Then, StringTie was used to identify new genes and calculated the expression value RPKM (Reads Per Kilobase of transcript per Million fragments mapped) for each gene [59]. PCA was performed in R v. 4.0.2 using the prcomp package. Differential expression analysis of two conditions was performed using the DESeq2. The resulting P values were adjusted using the Benjamini and Hochberg’s approach for controlling the false discovery rate. Genes with the absolute value of log_2_(fold change) ≥1 an adjusted *P*-value < 0.05 found by DESeq2 were assigned as differentially expressed genes (DEGs). Gene Ontology (GO) enrichment analysis of the DEGs was implemented by the GOseq R packages based Wallenius non-central hyper-geometric distribution [60]. The software KOBAS 2.0 [61] was used to test the statistical enrichment of differential expression genes in KEGG pathways.

### Preparation of metabolic samples

Rice metabolites from leaf samples were extracted as described previously [62]. In brief, freeze-dried leaf samples were ground into powder using a mixer mill (MM400, Retsch) for 1.5 min at 30 Hz. Powder (100 mg) was weighed and extracted overnight at 4°C with 0.8 ml 70% aqueous methanol (methanol:H_2_O_2_, 70:30, v/v) and pure methanol, respectively, followed by centrifugation for 10 min at 10 000 g. The supernatants were collected separately and combined together, followed by filtration with a microporous membrane (0.22 μm) before LC-MS analysis. The samples were equally mixed into multiple quality control samples (QC1, QC2 and QC3) for testing instrument stability.

### Metabolome analysis

The sample extracts were analyzed using an UPLC-ESI-MS/MS system (UPLC, ExionLC AD; MS, Applied Biosystems 4500 Q TRAP). The analytical conditions were as follows, UPLC: column, Agilent SB-C18 (1.8 μm, 2.1 mm * 100 mm); The mobile phase was consisted of solvent A, pure water with 0.1% formic acid, and solvent B, acetonitrile with 0.1% formic acid. Sample measurements were performed with a gradient program that employed the starting conditions of 95% A, 5% B. Within 9 min, a linear gradient to 5% A, 95% B was programmed, and a composition of 5% A, 95% B was kept for 1 min. Subsequently, a composition of 95% A, 5.0% B was adjusted within 1.1 min and kept for 2.9 min. The flow velocity was set as 0.35 mL per minute; The column oven was set to 40°C; The injection volume was 4 μL. The effluent was alternatively connected to an ESI-triple quadrupole-linear ion trap (QTRAP)-MS.

The ESI source operation parameters were as follows: source temperature 550°C; ion spray voltage (IS) 5500 V (positive ion mode)/-4500 V (negative ion mode); ion source gas I (GSI), gas II(GSII), curtain gas (CUR) were set at 50, 60, and 25 psi, respectively; the collision-activated dissociation(CAD) was high. QQQ scans were acquired as MRM experiments with collision gas (nitrogen) set to medium. DP (declustering potential) and CE (collision energy) for individual MRM transitions was done with further DP and CE optimization. A specific set of MRM transitions were monitored for each period according to the metabolites eluted within this period.

Metabolite identification is based on the precise molecular mass, MS2 fragments, isotopic distributions of MS2 fragments, and retention time (RT). The secondary spectra and RT of metabolites from samples are systematically and accurately matched one by one against the corresponding secondary spectra and RT in database. The mass tolerance for both MS and MS2 is set at 20 ppm, and the RT tolerance is set at 0.2 min. The database used in this study is a self-constructed database of Metware Biotechnology Inc. (Metware Database, MWDB).

Unsupervised PCA (principal component analysis) was performed by statistics function prcomp within R (www.r-project.org). The data was unit variance scaled before unsupervised PCA.

For two-group analysis, differential accumulated metabolites (DAMs) were determined by VIP (VIP ≥ 1) and absolute Log_2_FC(|Log_2_FC| ≥ 1.0). VIP values were extracted from OPLS-DA result, which also contain score plots and permutation plots, was generated using R package MetaboAnalystR. The data was log transform (log_2_) and mean centering before OPLS-DA. In order to avoid overfitting, a permutation test (200 permutations) was performed. Metabolite–metabolite interaction networks was extracted from STITCH 5 [27].

### Gene expression verification and fungal biomass analysis

The transcription levels of rice genes after inoculation were analyzed using qRT-PCR. Total RNA from *Pst*- or mock- inoculated rice leaves at 0, 6, 12, 24, 36, 48, 72, and 120 hpi was isolated using the Quick RNA extraction kit (Huayueyang Biotechnology, China, Beijing) according to the manufacturer’s protocol. The first-strand cDNA was synthesized with 1 μg total RNA using the HiScript II Q RT SuperMix for qPCR (Vazyme, Nanjing, China), and RT-PCR was performed on a Real-Time PCR Detection System (Bio-Rad, USA) using SYBR Premix reagent Q311 (Vazyme). *OsActin1* was used as the internal reference gene. The expression measurements were obtained according to the comparative 2^-ΔΔCT^ method.

Fungal biomass was analyzed by absolute quantitative PCR as previously reported [63]. DNA was collected and extracted from inoculated leaves at 14 dpi. Recombinant plasmids carrying *OsActin1* (*LOC_Os03g50885*) or *PstEF1* was used to establish standard curves of rice and *Pst*, and the number of gene copy was calculated by the standard curves. The primers for qRT-PCR are listed in S11 Table.

### Histological analysis of fungal growth

To observe the difference of fungal growth on mutant and WT rice plants, the *Pst*-inoculated rice leaves were sampled at 14 dpi. Leaf segments were fixed and decolorized in ethanl/ acetic acid (1:1, v/v). These leaves are then transferred to a saturated chloral hydrate solution and left overnight until the leaves are completely transparent. Leaves and fungi were stained with Calcofluor White (Sigma-Aldrich) as previously described [64]. In order to obtain high quality image of *Pst* infection structure in rice leaf tissue, the infected leaves were stained with wheat germ agglutinin (WGA) conjugated to Alexa-488 (Invitrogen) as previously described [65]. The infection area were observed with an Olympus BX-53 microscope and measured using the cellSens Entry software (Olympus).

### Phloroglucinol staining for lignin

Lignin accumulation at fungal infection sites in leaves was assessed using the phloroglucinol-HCl staining method, as previously described [66], with minor modifications. Leaf slices were hand-cut from calcofluor-stained samples and stained with a 3% phloroglucinol solution (Adamas) prepared in concentrated HCl (37N). Lignin deposition was visualized using an Olympus BX-53 microscope equipped with differential interference contrast (DIC) optics.

### Quantification of lignin and monolignols by GC-MS analysis

Cell wall residues (CWRs) for lignin analysis were prepared from inoculated rice leaves at 0 and 2 days post-inoculation (dpi) as previously described [67]. Briefly, frozen and dried leaf samples were pulverized by a TissueLyser for 5 minutes at 30Hz. The powder obtained was sequentially extracted with methanol, hexane and distilled water, followed by freeze-drying to obtain the CWRs.

Thioacidolysis of CWRs was performed as described [68]. The 10 mg of CWRs were weighed and transferred into a 2 ml brown glass vial. To each vial, 1 ml of freshly prepared reagent (dioxane: boron trifluoride diethyl etherate: ethanethiol = 175: 5: 20, v/v/v) was added. Vials were heated at 95°C for 4 h, with hourly manual agitation. After heating, samples were placed on ice for 15 minutes to terminate the reaction, followed by the addition 0.4 M NaHCO_3_ (about 300 μL) to adjust the pH to 3–4. The reaction mixture was transferred to a 10 ml tube, and the 2 ml vials were rinsed with 2 ml of water and 1 ml of dichloromethane. The washing liquid was transferred to the 10 ml tube. The mixture was vortexed, keep for 0.5 h, and centrifuged at 3000 rpm for 10 minutes. The organic phase was transferred to a 2 ml tube, and evaporated at 45°C. The dried residue was resuspended in 0.5 ml of dichloromethane. For derivatization, 100 μL of the resuspended sample was mixed with 50 μL of pyridine and 50 μL of *N,O*-bis(trimethylsilyl) acetamide. After incubation for at least 2 h at RT, 1 μL of this reaction product was analyzed by GC-MS.

An Agilent gas chromatography-mass spectrometry (GC–MS) was equipped with a DB-5 column (30 m × 0.25 mm × 0.25 mm). The inlet was held at 270°C, and the transfer line to the MS was held at 260°C. The following temperature gradient is used: initial hold at 130°C for 3 minutes; a 10°C/ min ramp to 200°C; a 3°C/ min ramp to 260°C and hold for 5 minutes. The flow rate was set at 1 ml/min. Peaks are identified by characteristic mass spectrum ions of 299 m/z, 269 m/z, and 239 m/z for S, G, and H monomers, respectively.

## Supporting information

S1 Fig KEGG pathway enrichment analysis of rice genes upregulated during *Puccinia striiformis* f. sp. *tritici* (*Pst*) infection. Dot plot shows the up-regulated KEGG pathways enriched for 24 hours post inoculation (hpi) **(A)**, 48 hpi **(B)** and 120 hpi **(C)**. The size of the dot is based on gene count enriched in the pathway, and the color of the dot shows the pathway enrichment significance.
(PDF)

S2 Fig Mapman analysis of differentially expressed genes. The distribution of DEGs from infected rice plants at 24 hpi **(A)**, 48 hpi **(B)** and 120 hpi **(C)** among various cellular processes, visualized by MapMan. The intensity of the color indicates the level of differential expression. Scale bar displays log2(fold change) values. Red and blue colors represent up-and down-regulation, respectively.
(PDF)

S3 Fig Principal Component analysis (PCA) plot of the metabolomics data. PC1 and PC2 represent the first and second principal components, respectively. The percentages indicate the proportion of total variance explained by each principal component. Each point in the plot represents an individual sample, with samples from the same group displayed in the same color.
(PDF)

S4 Fig Analysis of coefficient of variation (CV) for metabolomic data from different samples. The x-axis represents the CV value, while the y-axis indicates the proportion of metabolites with a CV value less than the corresponding value relative to the total number of metabolites. Different colors represent different sample groups, with QC denoting quality control samples. The two vertical reference lines correspond to CV values of 0.3 and 0.5, while the two horizontal reference lines indicate proportions of 75% and 85% of the total number of metabolites.
(PDF)

S5 Fig Heatmap of Pearson correlation coefficient matrix. Correlation matrix showing the Pearson correlation coefficients (r) between different metabolomic dataset from samples at 0, 48, and 120 hours post inoculation with *Puccinia striiformis* f. sp. *tritici* (*Pst*) and mock control. The rows and diagonal represent the names of different samples. Colors indicate the magnitude and direction of the Pearson correlation coefficient: red shades denote stronger positive correlations, green shades indicate weaker correlations, and blue shades represent stronger negative correlations. The numerical values of the correlation coefficients are displayed within each cell for precise interpretation.
(PDF)

S6 Fig The number of differentially accumulated metabolites in different categories. (PDF)

S7 Fig KEGG pathway enrichment analysis of differentially accumulated metabolites in rice during *Puccinia striiformis* f. sp. *tritici* (*Pst*) infection. Dot plot shows the up-regulated KEGG pathways enriched for 48 hours post inoculation (hpi) **(A)** and 120 hpi **(B)**. The size of the dot is based on gene count enriched in the pathway, and the color of the dot shows the pathway enrichment significance.
(PDF)

S8 Fig Validation of the transcriptional induction of genes involved in lignin biosynthesis in rice infected by *Puccinia striiformis* f. sp. *tritici* (*Pst*) by qRT-PCR. Values represent means from three biological replicates, with error bars indicating the standard deviation (SD). Asterisks indicate statistically significant differences compared to samples at 0 hours post inoculation (hpi) with *Pst* using two-sided Student’s *t*-test. (*P < 0.05, **P < 0.01).
(PDF)

S9 Fig Schematic diagram of the sgRNAs targeting the gene *OsPAL3*, *Os4CL5*, *Os4CL3*, and *OsCCoAOMT* in rice mutants. (PDF)

S1 Table Alignment results from each RNA-Seq samples. (XLSX)

S2 Table Differentially expressed genes (DEGs) in rice during *Puccinia striiformis* f. sp. *tritici* (*Pst*) infection. (XLSX)

S3 Table KEGG enrichment of metabolic pathways by upregulated genes in rice upon *Puccinia striiformis* f. sp. *tritici* (*Pst*) infection. (XLSX)

S4 Table Metabolites identified by wide-targeted metabolomics analysis. (XLSX)

S5 Table KEGG enrichment analysis of differentially-accumulated metabolites. (XLSX)

S6 Table Expression patterns of rice peroxidase genes during *Puccinia striiformis* f. sp. *tritici* (*Pst*) infection. (XLSX)

S7 Table Expression patterns of rice laccase genes during *Puccinia striiformis* f. sp. *tritici* (*Pst*) infection. (XLSX)

S8 Table Infection site areas at 14 dpi (in units of 1000 µm^2^). (XLSX)

S9 Table Biomass ratio (*Pst*/rice) of total DNA extracted from wild-type and mutant rice leaves at 14 dpi. (XLSX)

S10 Table Relative quantification of H-, G-, and S-units of lignin in wild-type and mutant rice leaves. (XLSX)

S11 Table Primer list used in this study. (XLSX)
