# Supplementary material for: Coordinated transcriptomic and metabolomic responses in rice reveal lignin-based physical barriers as key mechanisms of nonhost resistance to rust fungi
Source: PLoS Genet. 2025 May 9;21(5):e1011679. doi: 10.1371/journal.pgen.1011679 (PMC12121910; doi:10.1371/journal.pgen.1011679)
Supplement: S1 Fig — Dot plot shows the up-regulated KEGG pathways enriched for 24 hours post inoculation (hpi) (A), 48 hpi (B) and 120 hpi (C). The size of the dot is based on gene count enriched in the pathway, and the color of the dot shows the pathway enrichment significance. (PDF) [file pgen.1011679.s001.pdf]

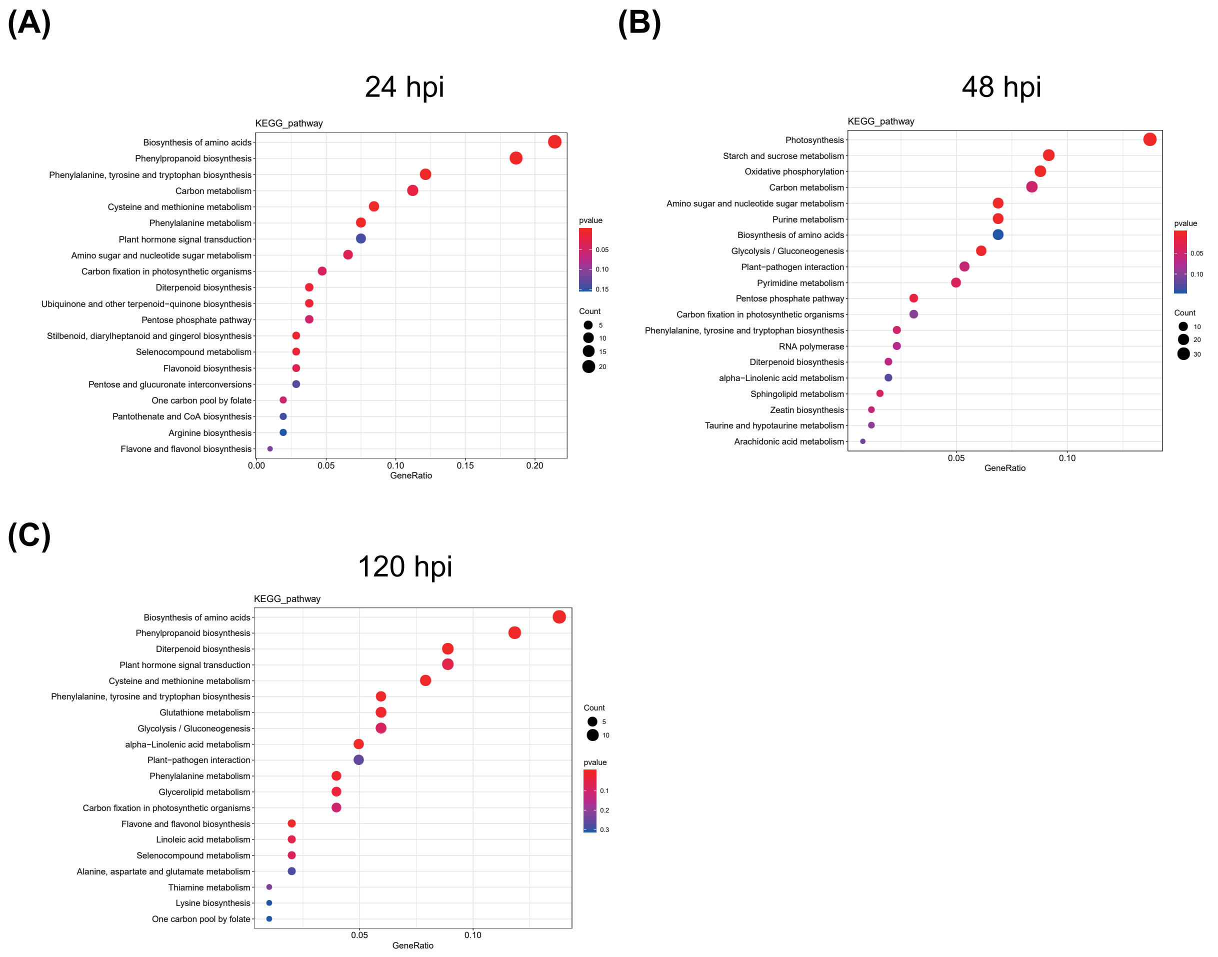

S1 Fig. KEGG pathway enrichment analysis of rice genes up-regulated during *Puccinia striiformis* f. sp. *tritici* (*Pst*) infection. Dot plot shows the up-regulated KEGG pathways enriched for 24 hpi (A), 48 hpi (B) and 120 hpi (C). The size of the dot is based on gene count enriched in the pathway, and the color of the dot shows the pathway enrichment significance.
