# Supplementary material for: Coordinated transcriptomic and metabolomic responses in rice reveal lignin-based physical barriers as key mechanisms of nonhost resistance to rust fungi
Source: PLoS Genet. 2025 May 9;21(5):e1011679. doi: 10.1371/journal.pgen.1011679 (PMC12121910; doi:10.1371/journal.pgen.1011679)
Supplement: S2 Fig — The distribution of DEGs from infected rice plants at 24 hpi (A), 48 hpi (B) and 120 hpi (C) among various cellular processes, visualized by MapMan. The intensity of the color indicates the level of differential expression. Scale bar displays log2(fold change) values. Red and blue colors represent up-and down-regulation, respectively. (PDF) [file pgen.1011679.s002.pdf]

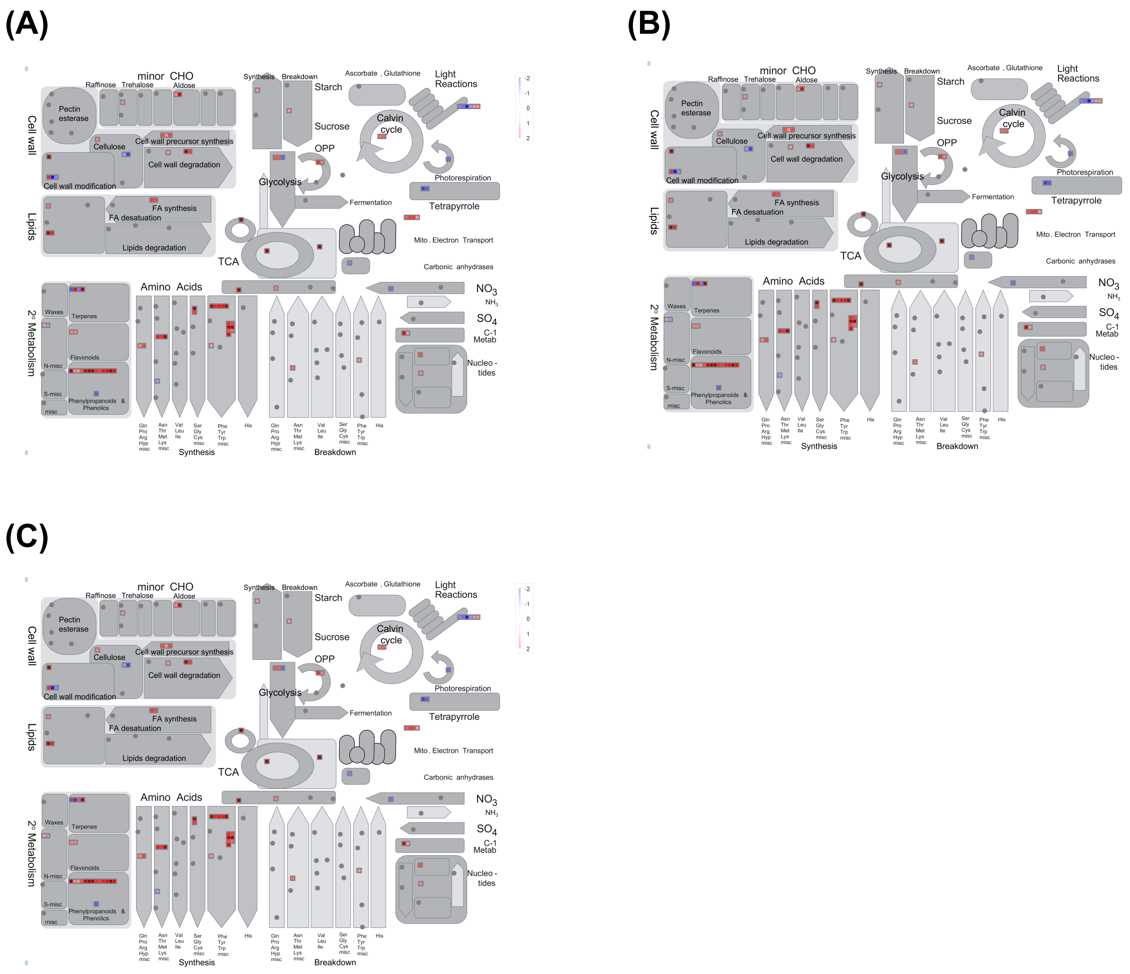

S2 Fig. Mapman analysis of differentially expressed genes. The distribution of DEGs from infected rice plants at 24 hpi **(A)**, 48 hpi **(B)** and 120 hpi **(C)** among various cellular processes, visualized by MapMan. The intensity of the color indicates the level of differential expression. Scale bar displays log2(fold change) values. Red and blue colors represent up-and down-regulation, respectively.
