# Supplementary material for: Coordinated transcriptomic and metabolomic responses in rice reveal lignin-based physical barriers as key mechanisms of nonhost resistance to rust fungi
Source: PLoS Genet. 2025 May 9;21(5):e1011679. doi: 10.1371/journal.pgen.1011679 (PMC12121910; doi:10.1371/journal.pgen.1011679)
Supplement: S3 Fig — PC1 and PC2 represent the first and second principal components, respectively. The percentages indicate the proportion of total variance explained by each principal component. Each point in the plot represents an individual sample, with samples from the same group displayed in the same color. (PDF) [file pgen.1011679.s003.pdf]

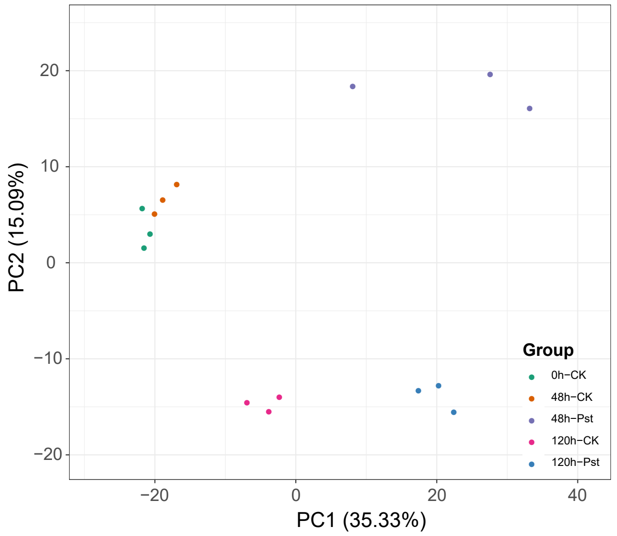

S3 Fig. Principal Component analysis (PCA) plot of the metabolomics data. PC1 and PC2 represent the first and second principal components, respectively. The percentages indicate the proportion of total variance explained by each principal component. Each point in the plot represents an individual sample, with samples from the same group displayed in the same color.
