# Supplementary material for: Coordinated transcriptomic and metabolomic responses in rice reveal lignin-based physical barriers as key mechanisms of nonhost resistance to rust fungi
Source: PLoS Genet. 2025 May 9;21(5):e1011679. doi: 10.1371/journal.pgen.1011679 (PMC12121910; doi:10.1371/journal.pgen.1011679)
Supplement: S4 Fig — The x-axis represents the CV value, while the y-axis indicates the proportion of metabolites with a CV value less than the corresponding value relative to the total number of metabolites. Different colors represent different sample groups, with QC denoting quality control samples. The two vertical reference lines correspond to CV values of 0.3 and 0.5, while the two horizontal reference lines indicate proportions of 75% and 85% of the total number of metabolites. (PDF) [file pgen.1011679.s004.pdf]

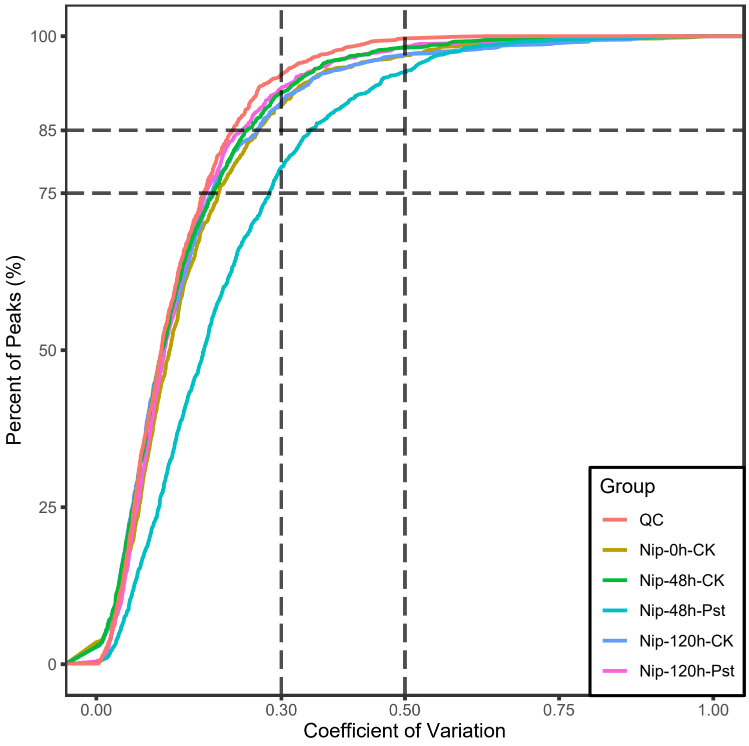

S4 Fig. Analysis of coefficient of variation (CV) for metabolomic data from different samples. The x-axis represents the CV value, while the y-axis indicates the proportion of metabolites with a CV value less than the corresponding value relative to the total number of metabolites. Different colors represent different sample groups, with QC denoting quality control samples. The two vertical reference lines correspond to CV values of 0.3 and 0.5, while the two horizontal reference lines indicate proportions of 75% and 85% of the total number of metabolites.
