# Supplementary material for: Coordinated transcriptomic and metabolomic responses in rice reveal lignin-based physical barriers as key mechanisms of nonhost resistance to rust fungi
Source: PLoS Genet. 2025 May 9;21(5):e1011679. doi: 10.1371/journal.pgen.1011679 (PMC12121910; doi:10.1371/journal.pgen.1011679)
Supplement: S5 Fig — Correlation matrix showing the Pearson correlation coefficients (r) between different metabolomic dataset from samples at 0, 48, and 120 hours post inoculation with Puccinia striiformis f. sp. tritici (Pst) and mock control. The rows and diagonal represent the names of different samples. Colors indicate the magnitude and direction of the Pearson correlation coefficient: red shades denote stronger positive correlations, green shades indicate weaker correlations, and blue shades represent stronger negative correlations. The numerical values of the correlation coefficients are displayed within each cell for precise interpretation. (PDF) [file pgen.1011679.s005.pdf]

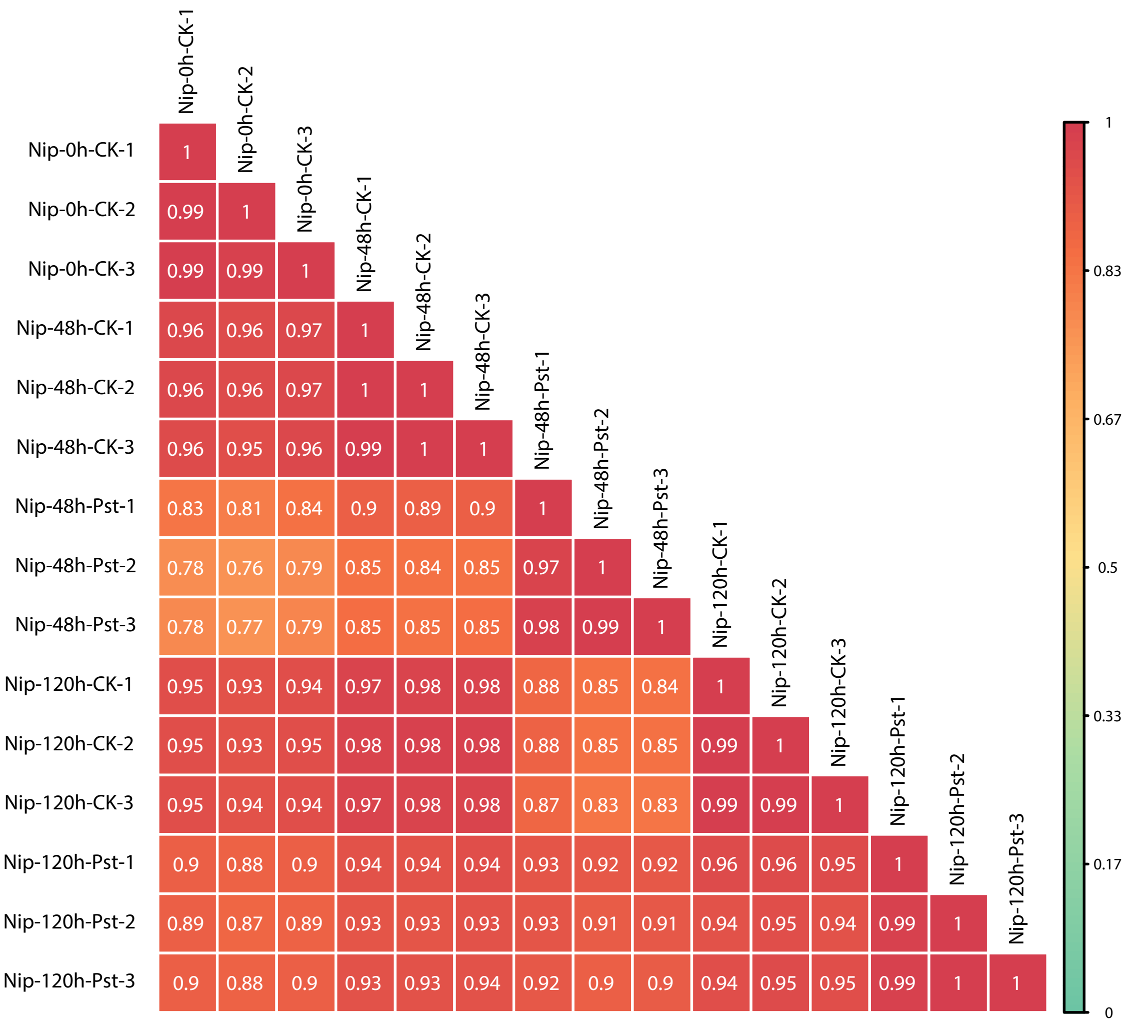

S5 Fig. Heatmap of Pearson correlation coefficient matrix. Correlation matrix showing the Pearson correlation coefficients (r) between different metabolomic dataset from samples at 0, 48, and 120 hours post inoculation with *Puccinia striiformis* f. sp. *tritici* (*Pst*) and mock control. The rows and diagonal represent the names of different samples. Colors indicate the magnitude and direction of the Pearson correlation coefficient: red shades denote stronger positive correlations, green shades indicate weaker correlations, and blue shades represent stronger negative correlations. The numerical values of the correlation coefficients are displayed within each cell for precise interpretation.
