# Supplementary figures and images for: Coordinated transcriptomic and metabolomic responses in rice reveal lignin-based physical barriers as key mechanisms of nonhost resistance to rust fungi
Source: PLoS Genet. 2025 May 9;21(5):e1011679. doi: 10.1371/journal.pgen.1011679 (PMC12121910; doi:10.1371/journal.pgen.1011679)

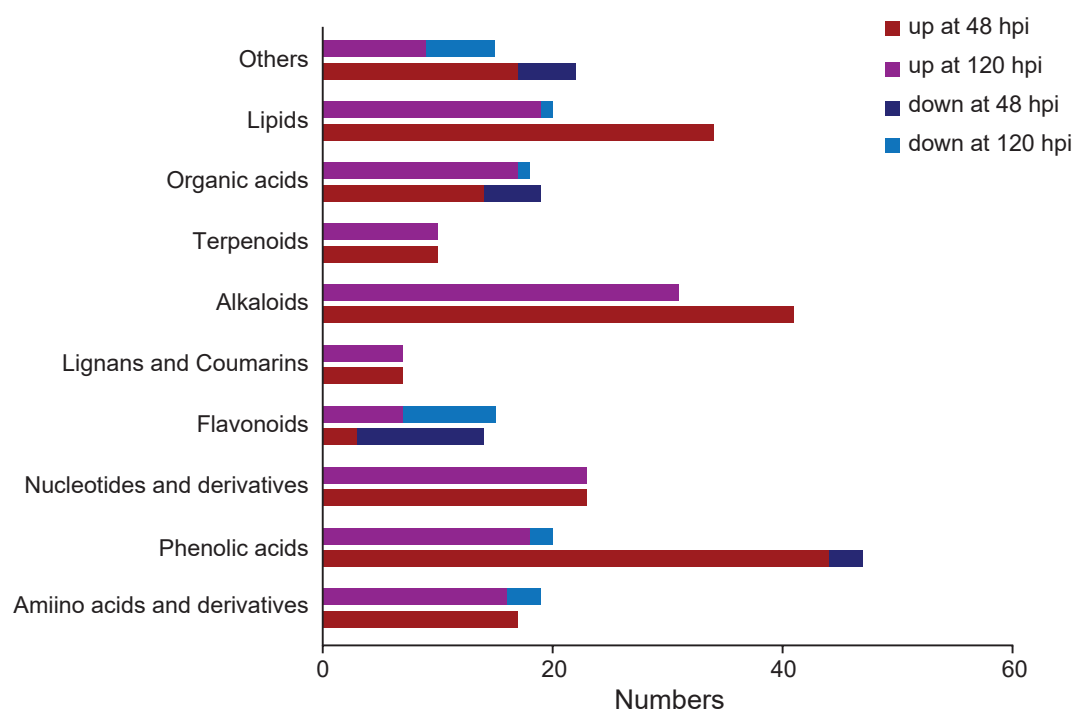

**S6 Fig.** The number of differentially accumulated metabolites in different categories.

Supplement: S6 Fig — (PDF) [file pgen.1011679.s006.pdf]
