# Supplementary material for: Coordinated transcriptomic and metabolomic responses in rice reveal lignin-based physical barriers as key mechanisms of nonhost resistance to rust fungi
Source: PLoS Genet. 2025 May 9;21(5):e1011679. doi: 10.1371/journal.pgen.1011679 (PMC12121910; doi:10.1371/journal.pgen.1011679)
Supplement: S8 Fig — Values represent means from three biological replicates, with error bars indicating the standard deviation (SD). Asterisks indicate statistically significant differences compared to samples at 0 hours post inoculation (hpi) with Pst using two-sided Student’s t-test. (*P < 0.05, **P < 0.01). (PDF) [file pgen.1011679.s008.pdf]

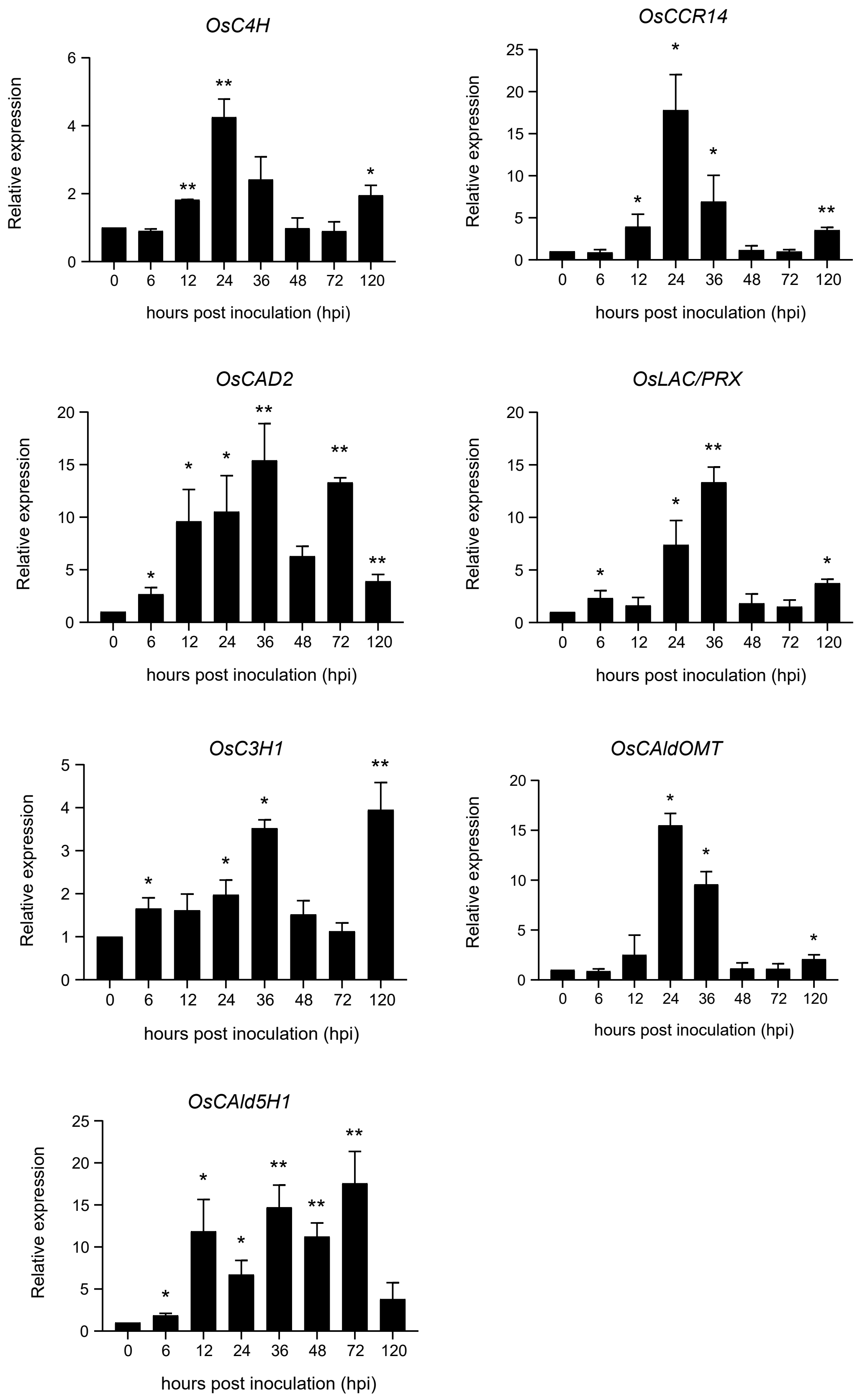


**S8 Fig. Validation of the transcriptional induction of genes involved in lignin biosynthesis in rice infected by *Puccinia striiformis* f. sp. *tritici* (*Pst*) by qRT-PCR.** Values represent means from three biological replicates, with error bars indicating the standard deviation (SD). Asterisks indicate statistically significant differences compared to samples at 0 hours post inoculation (hpi) with ***Pst*** using two-sided Student's *t*-test. (*$P$ <0.05, **$P$ < 0.01).
