# Supplementary material for: Coordinated transcriptomic and metabolomic responses in rice reveal lignin-based physical barriers as key mechanisms of nonhost resistance to rust fungi
Source: PLoS Genet. 2025 May 9;21(5):e1011679. doi: 10.1371/journal.pgen.1011679 (PMC12121910; doi:10.1371/journal.pgen.1011679)
Supplement: S9 Fig — (PDF) [file pgen.1011679.s009.pdf]

***LOC_Os02g41670* (*OsPAL3*)**

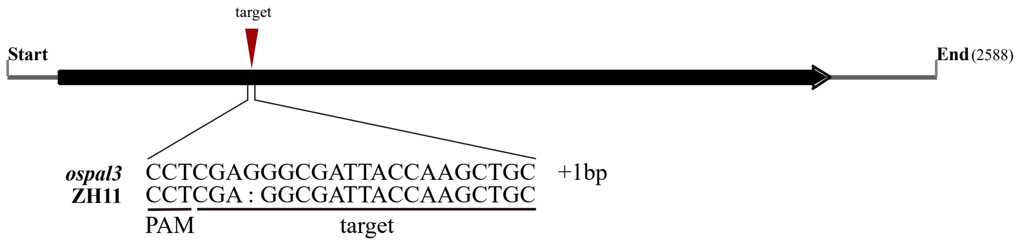

***LOC_Os08g34790* (*Os4CL5*)**

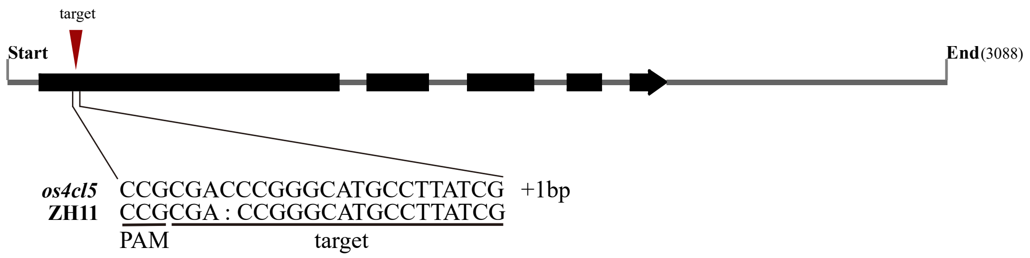

***LOC_Os02g08100* (*Os4CL3*)**

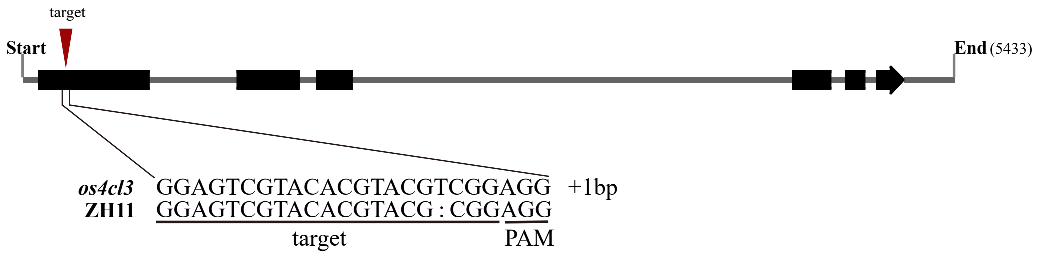

***LOC_Os08g38900* (*OsCCoAOMT*)**

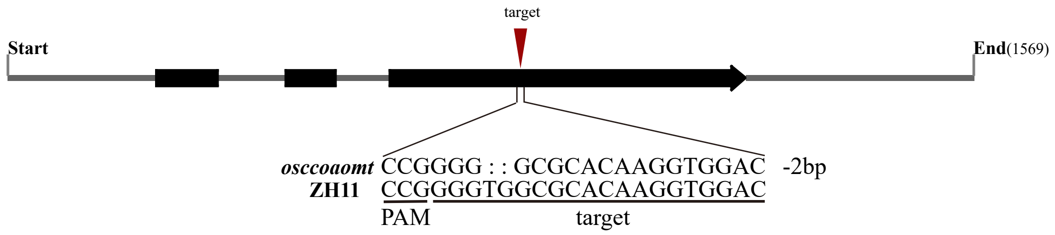

S9 Fig. Schematic diagram of the sgRNAs targeting genes *OsPAL3, Os4CL5, Os4CL3*, and *OsCCoAO-MT* in rice mutants.
